# Development of flank lesions in growing pigs after weaning: A case study

**DOI:** 10.3389/fvets.2022.1070206

**Published:** 2023-01-12

**Authors:** Marianna Norring, Heng-Lun Ko, Anna Valros

**Affiliations:** ^1^Research Centre for Animal Welfare, Department of Production Animal Medicine, University of Helsinki, Helsinki, Finland; ^2^Department of Animal and Food Science, School of Veterinary Science, Universitat Autònoma de Barcelona, Cerdanyola del Vallès, Spain

**Keywords:** damaging behavior, flank lesion, injurious behavior, skin lesion, Sus scrofa

## Abstract

Flank lesions in pigs are a common yet poorly understood consequence of damaging social behavior. One group of pigs on a commercial farm with group lactation and late weaning, and with the history of flank lesions was studied. Skin lesions on the flanks, including linear and circular lesions, and tail lesions on 69 pigs were recorded six times during 5 weeks after weaning at the age of 9 weeks. Nosing behavior was scanned during six sessions with multiple scans. The associations of age, trunk whiteness, weight gain, sow parity, litter size, sex, and tail lesions with the number of circular and linear lesions were analyzed using linear mixed models. The number of linear lesions increased as pigs aged, and pigs with a higher weight gain had more linear lesions. Moreover, pigs with a whiter trunk color were scored with more lesions of both types. According to descriptive behavior data, nosing and biting behaviors were most frequent during weeks 2–4 after weaning at the age of 11–13 weeks. On average, seven circular flank lesions were found per pig during the experiment, at the age of 10–14 weeks. After the peak on day 17, their occurrence decreased. Skin lesion occurrence was related to a lighter skin color on the trunks of pigs. We recommend reporting skin color in connection with lesion scoring results. Nosing behavior and flank lesions both peaked from 2 to 4 weeks after weaning, suggesting that nosing behavior contributed to lesion development during this time.

## 1. Introduction

A survey conducted in the UK reported that farmers ranked the tail, ear and flank biting as the most important welfare issue to be tackled in the grower and finisher stage (1). According to a Danish investigation, ear necrosis and tail and flank bites, accounted for 43% of the clinical signs reported on finisher pigs (2). While damaging behaviors are considered as a serious welfare issue *per se* (3), the lesions caused by these behaviors may also be an economic loss for farmers (4). While extensive information is available on some damaging behaviors, especially on tail and ear biting, less is known about the development of flank lesions.

Flank biting behavior in pigs, however, appears to be common, and flank-directed behavior was noted in almost all of the 31 finishing pig farms in an Irish study (5). However, the onset and the peak of flank-nosing or flank-biting behavior seem to vary: Straw and Bartlett (6) observed a peak of nosing behaviors, including flank and belly nosing about 1-week post-weaning in piglets weaned at 3–4 weeks of age. van Staaveren et al. (7) observed that flank lesions were more prevalent at older ages (8–13 weeks and in finishers) than in 4–8 week-old pigs. Chou et al. (8) observed a peak in the frequency of damaging behaviors at 20 weeks of age in finisher pigs. The histology of flank biting lesions was described by Mirt (9), who suggested that they might be caused by a similar pathogenesis to ear lesions, whereby a slight trauma of the skin, for example, due to manipulation by another pig, allows infection with staphylococci. However, the etiology or causes are not well described or understood. van Staaveren et al. (5) found that mild and severe flank lesions existed together. In the same study, flank-directed behaviors were not correlated with flank lesion prevalence, while tail and ear directed behaviors and corresponding lesions were.

In general, damaging behaviors such as ear biting and tail biting often appear simultaneously in the same individuals (10, 11). Furthermore, skin lesions in general have been correlated with flank lesion prevalence on farms (7). Similar risk factors might influence these different damaging behaviors: pigs on farms using chains as enrichment objects displayed more damaging behaviors overall, as well as flank biting, compared to pigs on farms using wooden or plastic devices (5).

This case study aimed at describing the development of pig skin lesions in a group of growing pigs as an attempt to understand the etiology of flank biting. In addition, we observed nosing behaviors during the same period to assess whether these were temporally linked to lesion occurrence.

## 2. Materials and methods

### 2.1. Animals and housing

**Figure 1:**
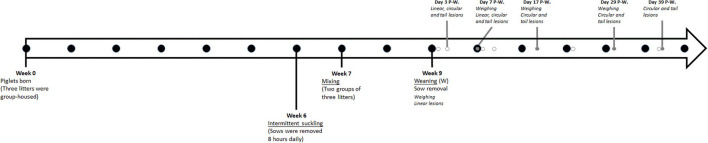
Timeline of the on-farm management (from Week 0 to 9) and the experimental design (from Week 9 to 15). The black dots indicate the week of the age of the piglets. The gray dots indicate the days post-weaning (P-W.) in which we carried out the experimental work, including piglet weighing or the assessment of linear/circular/tail lesions on piglets. The white dots indicate the days for behavioral observation (days 1, 3, 8, 10, 22, and 38 post-weaning).

The study was performed from February to March 2018 on a commercial farm located in Southern Finland with a history of flank lesions. According to the farmer, the occurrence of flank lesions was similar to other groups of weaned pigs on the farm. Sixty-nine weaners from six litters (mean litter size: 12 ± 4) were born in a group-farrowing system. The sows were Landrace and Landrace × Yorkshire mixed breeds. Two groups of three sows and their offspring were kept separately for the first 6 weeks. During the 5th week after farrowing, the sows were removed daily for about 8 h to promote estrus. On the 6th week after farrowing, these two groups were mixed together into a group pen where this study was performed. The sows were finally removed when the piglets reached the age of 9 weeks (i.e., weaning). The study commenced after weaning and continued for 6 weeks (Figure 1).

**Figure 2:**
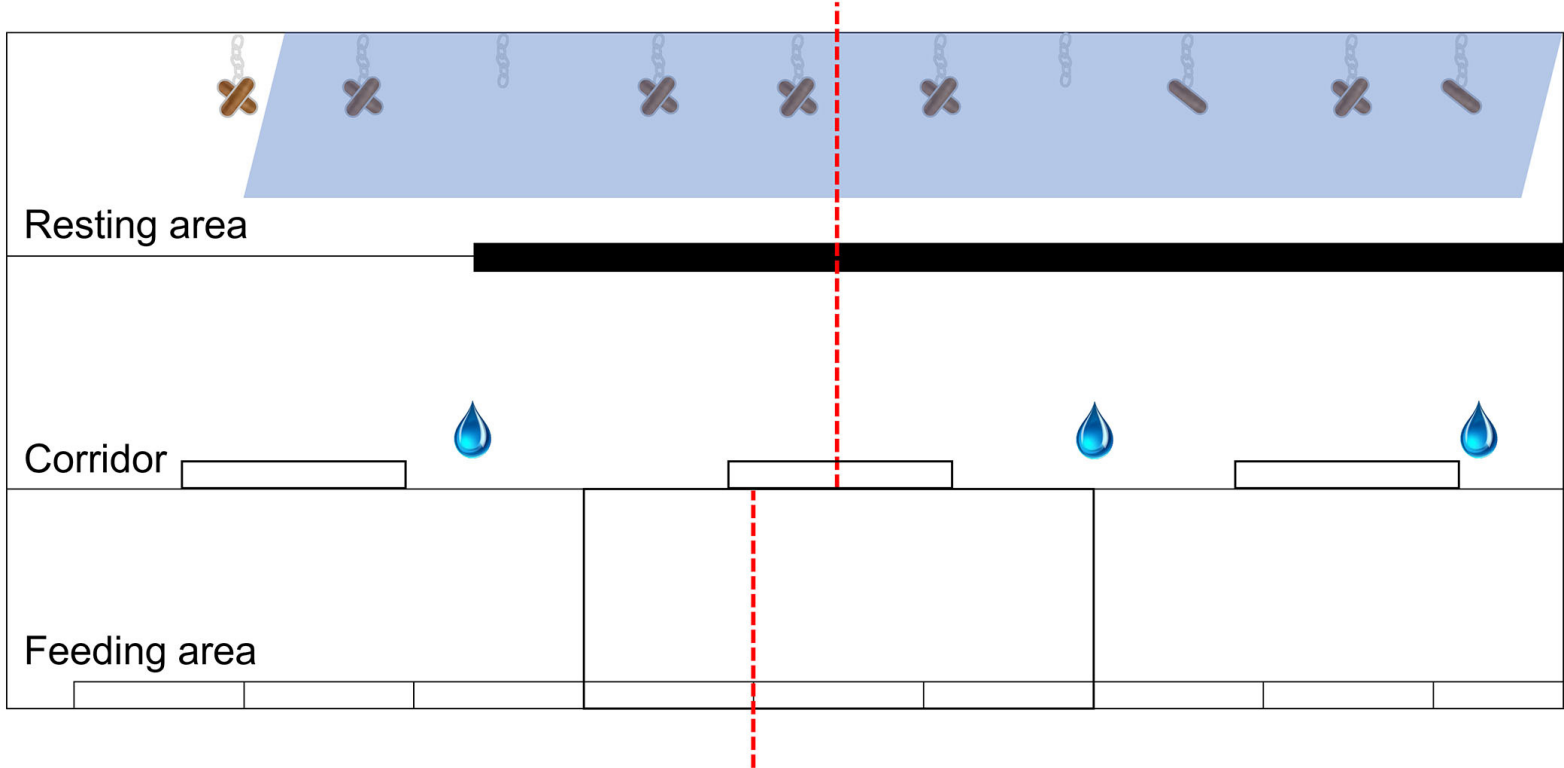
Schematic diagram illustrating the three areas in the growing pen, including the feeding area (17.4*2.5 m), corridor (17.4*2.6 m), and resting area (17.4*2.5 m). The blue shaded trapezoid in the resting area indicates the roof cover. Several wooden logs were hung from the wall by metal chains as drawn in the resting area. The narrow black rectangle indicates the gates where pigs could not pass between the resting area and corridor. The white rectangles in the corridor and feeding area indicate the feed troughs. The water droplets indicate the location of nipple drinkers. The red dashed lines indicate the division of the pen into separate observation sectors for live behavioral observation (feeding 1, corridor 1, and resting 1 to the right; feeding 2, corridor 2, and resting 2 to the left).

The pigs were fed three times a day (at 08:00, 16:00, and 21:00) a diet following Finnish recommendations. Water was provided *ad libitum*. In the growing pen, there were three designated areas, all with solid concrete flooring: a feeding area (17.4^*^2.5 m), corridor (17.4^*^2.6 m), and resting area (17.4^*^2.5 m) (Figure 2). There were nine feed troughs in the feeding area, as well as three additional feed troughs and six nipple drinkers in the corridor. The roof-covered resting area was straw-bedded, and several wooden logs hung from the wall by chains. Straw bedding was renewed weekly. The room temperature was maintained at ~19 ± 2°C.

### 2.2. Procedures and skin lesion assessment

For another experiment, piglets had been ear-tagged after birth using different colors for each litter. All the pigs were weighed on the day of weaning (9 weeks of age), as well as 7 days (10 weeks of age), 17 days (13 weeks of age), and 29 days (14 weeks of age) post-weaning. While the pigs were on the scale, lesions were closely observed with a hand-held light source. Two trained observers were used to score the lesions. Linear lesions were scored on the day of weaning and 3 and 7 days post-weaning. As the current study focused on circular lesions, circular lesions and tail lesions were assessed on the day of weaning and 3, 7, 17, 29, and 39 days after weaning. Circular and linear lesions were observed on sides and back, excluding head, neck and belly areas.

The number and severity of circular lesions were scored according to a scoring system modified from Diana et al. (12) (Table 1). The weighted number of circular lesions was calculated by multiplying the number of severe lesions (Table 1) by two and summing it with number of minor circular lesions to better describe the overall severity and affected skin area and because only few severe lesions were found.

**Table 1:** Scoring system for the circular, linear, and tail lesions in pigs [adapted from (12)].

**Lesion type**	**Description**
Minor circular lesion	A minor (superficial, diameter: ≥1 cm) circular lesion.
Severe circular lesion	A larger (diameter: ≥2 cm), or deep circular lesion and/or blood and/or infection on the flank.
Linear lesion	Red and/or swollen fresh linear scratches.
Mild tail lesion	Redness of the skin or minor scratches.
Severe tail lesion	An open wound, and/or a clear bite mark, and/or part of the tail missing and/or swollen lesion.

In addition, as some pigs were completely white or black and some had black spots, the trunk color was recorded as the estimated percentage of the total trunk (sides and back, excluding head, neck, and belly areas) that was white (i.e., trunk whiteness) during the first lesion observations.

### 2.3. Behavioral observations

Two previously trained observers scan sampled the pig group at 5-min intervals. As the whole group was not visible at once, the growing pen was divided into six observation sectors (two feeding sectors, two corridor sectors, two resting sectors, Figure 2). One observer scanned three sectors and the other observer simultaneously scanned the other three sectors (see the division in Figure 2). Behavior was observed for 4 h per day (between 9:00 and 15:00), resulting in 48 scans per day. Observations took place for 6 days (twice during weeks 1 and 2 and once during weeks 4 and 6 after weaning).

A biting event was defined as the focal pig biting the receiver's trunk, belly, or other body parts (including the neck, face, tail, and legs) once. Nosing was defined as the focal pig's snout continuously making physical contact with the receiver's trunk, belly, or other body parts. A minimum of three consecutive contacts with a certain body part (trunk/belly/other) were needed to be considered as the beginning of one nosing event. All pigs visible were scanned and if physical contact was observed this pig was followed to see if the contact continued. Nosing event was considered discontinued when 3 s elapsed without physical contact. Biting events very seldomly occurred and they were thus scored as combined with the more numerous nosing events.

The number of events was recorded per observation sector. Nosing and biting events per day were summed for data analysis, and weekly averages were calculated for the weeks with two observation days (weeks 1 and 2). As the pigs could not be identified from a distance, only descriptive data on behavior are reported.

### 2.4. Ethical statement

Pigs were managed according to normal farm practices under commercial conditions (13). No additional harm was expected to occur by handling and spectating, as the animals were moved with their usual group, and individual observation of skin lesions only took a few minutes. The study was licensed by the National Animal Experiment Board and complied with the national rules derived from European Directive 2010/63 on the protection of animals used for scientific purposes.

### 2.5. Statistical analysis

The associations of age, trunk whiteness, weight gain, sow parity, litter size, pig sex, and tail lesions with the number of circular lesions and weighted number of circular lesions were analyzed with linear mixed models. Age (6 levels) was used as repeated effect and pig was considered as the statistical unit. Weight gain, sow parity, litter size, pig sex, and tail lesions were not significantly (*P* > 0.05) related to numbers of circular lesions and were thus removed from the final model.

The associations of age, trunk whiteness, weight gain, sow parity, litter size, pig sex, and tail lesions with the number of linear lesions were analyzed with linear mixed models. Age (3 levels) was used as a repeated effect and pig was considered as the statistical unit. Sow parity, litter size, pig sex, and tail lesions were not significantly (*P* > 0.05) related to lesion numbers and were thus removed from the final model.

The chi-squared test was used to analyze the development of tail lesions after weaning. Only descriptive data were available for the development of behavior. Statistical analyses were performed with SPSS 24.

## 3. Results

### 3.1. Circular and linear lesions

**Figure 3:**
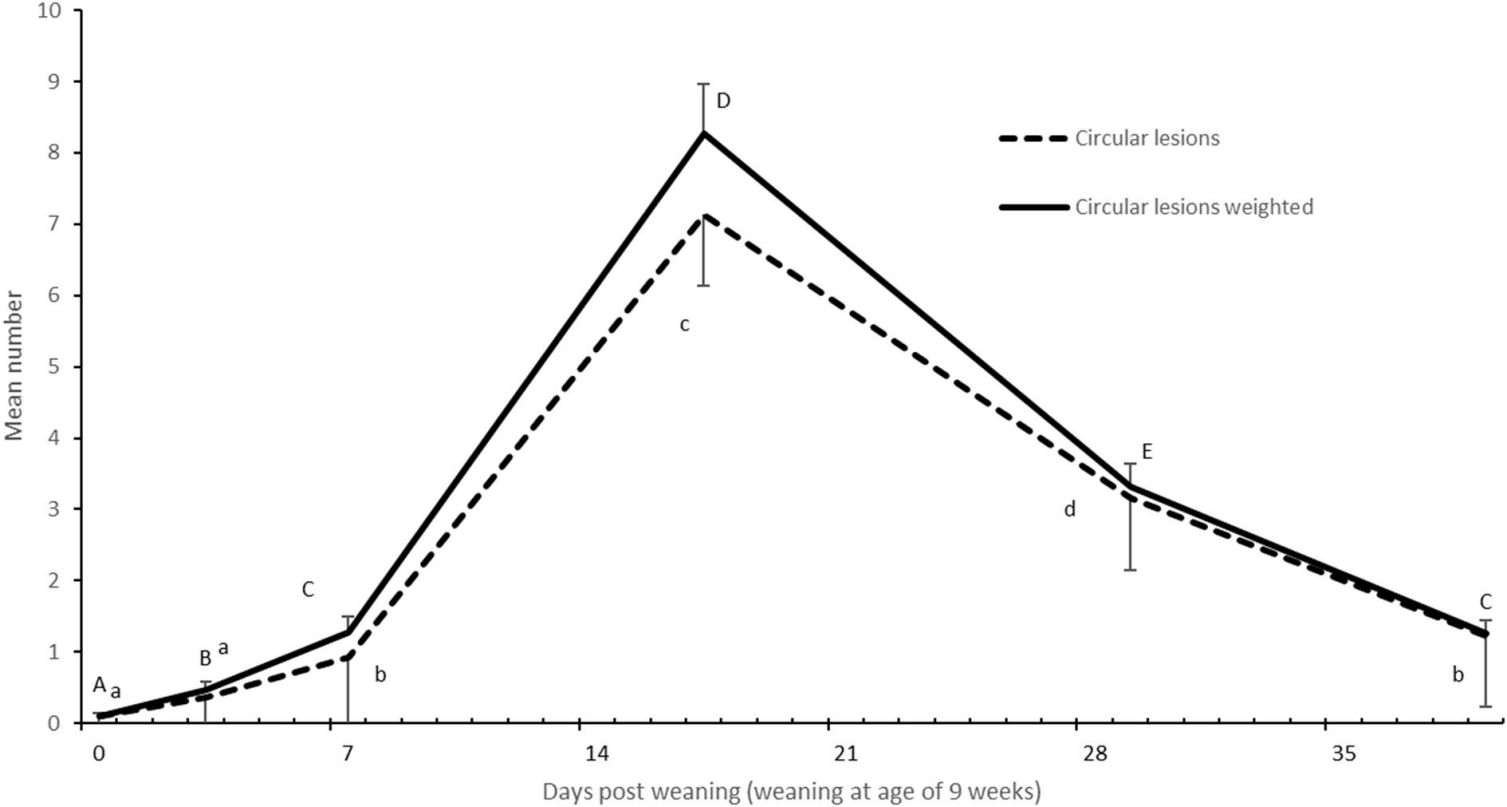
Development of the number of circular lesions (mean ± SE) per pig on the flanks of 69 pigs after weaning at 9 weeks of age. Means with different capital letters indicate a significant difference between days in the weighted number of circular lesions, and different lower-case letters indicate a significant difference between days in the number of circular lesions (*P* < 0.05). The weighted number of circular lesions was calculated by multiplying the number of severe lesions by two and summing it with the number of small lesions to reflect the size and severity of lesions.

The number of circular lesions on the flanks peaked on day 17 post-weaning (*P* = 0.001, Figure 3). The number of linear lesions on the flanks increased as pigs aged (*P* = 0.001). The number of linear lesions were: 9 ± 1.0^a^ on the day of weaning, 15 ± 1.4^b^ on day 3, and 18 ± 1.8^b^ on day 7 (^ab^different letter indicates difference). Pigs with a higher weight gain had more linear lesions (slope 26.3, *P* = 0.017).

Pigs with a whiter trunk color were scored with more circular lesions (slope 0.31, *P* = 0.023) and a higher weighted number of circular lesions (slope 0.30, *P* = 0.040). Moreover, pigs with a whiter trunk color were scored with more linear lesions (slope 9.9, *P* = 0.001).

### 3.2. Tail lesions

The majority of the pig tails were intact and no severe tail lesions were recorded. The occurrence of mild tail lesions differed significantly between days (*P* = 0.007). The number of mild tail lesions (total number of tails assessed) were: 18 (69) on the day of weaning, 6 (69) on day 3, 23(69) on day 7, 15 (64) on day 17, 12 (65) on day 29, and 9 (65) on day 39. However, no clear temporal pattern could be distinguished.

### 3.3. Biting and nosing behavior after weaning

**Figure 4:**
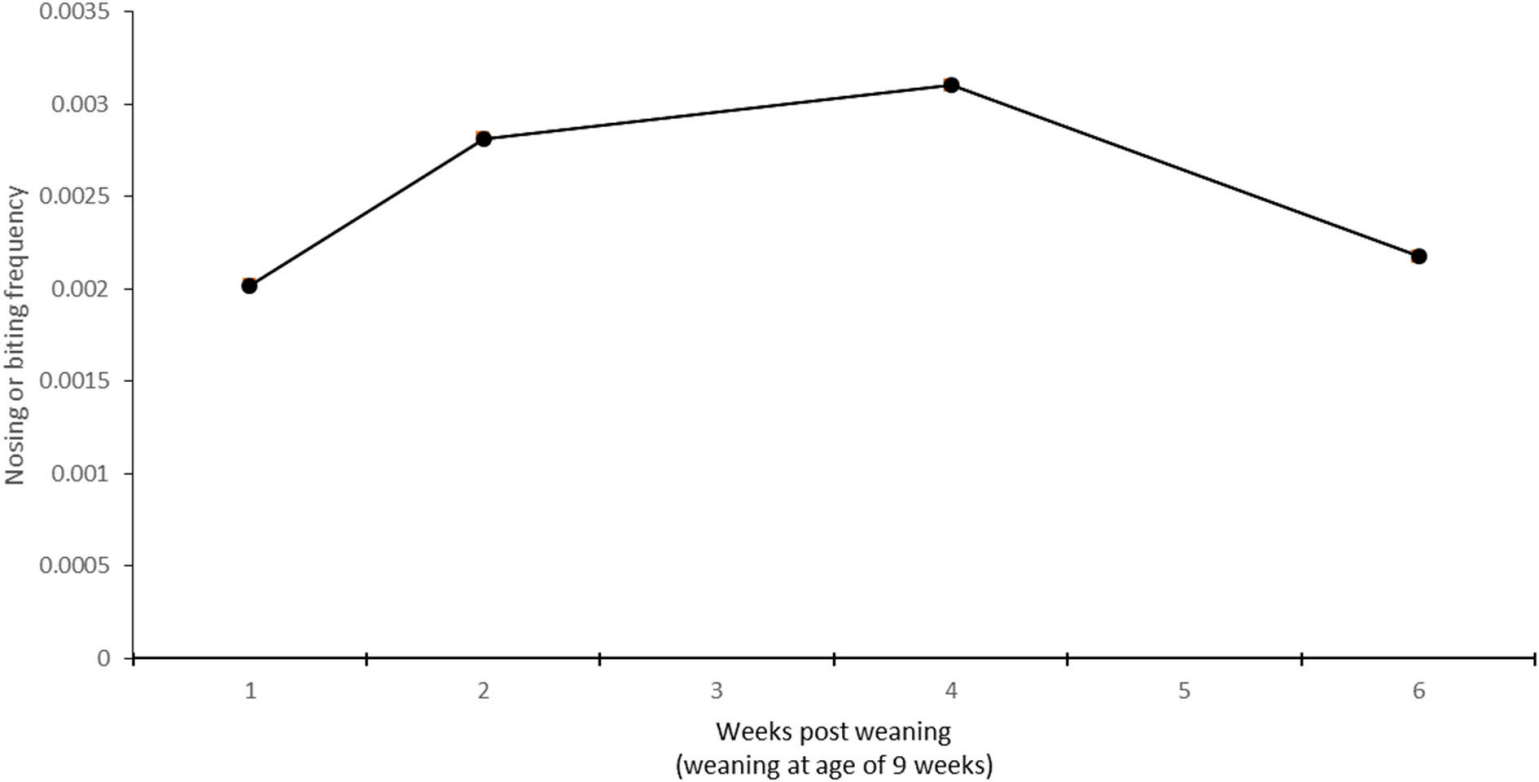
Frequency of nosing or biting behavior (directed to the trunk, belly, or other body parts) after weaning (weaning at 9 weeks of age) in one group of 69 pigs. Descriptive data based on weekly averages of 4 h instantaneous sampling (5-min interval), with observations during the daytime. Behaviors were observed twice during weeks 9 and 10 and once during weeks 12 and 14. Observed nosing events were divided by the number of animals visible in one of the three areas the pigs used for resting.

The development of the total frequency of nosing and biting behavior after weaning is illustrated in Figure 4. The descriptive data are based on the averages of 6 days (4 h of observations per day) of observations. The nosing and biting frequency are divided by the number of animals visible (as hourly averages) at any sampling time (5-min intervals). Figure 4 displays data from three resting areas only (resting 1, 2, and corridor 2, which had bedding), as very few nosing or biting behaviors were observed in the feeding and the unbedded corridor areas.

On average, nosing or biting of the belly area was observed the least, followed by the flank area, while other areas (including the neck, face, tail, and legs) attracted the highest frequency of nosing or biting events (belly 0.0004, flank 0.0017, other areas 0.0054 nosing events per 5-min scan sample divided by the number of pigs visible).

## 4. Discussion

Circular flank lesions increased after weaning, which occurred at the age of 9 weeks in the studied pigs. Circular flank lesions were most prominent on observation day 17 after weaning, after which their occurrence decreased. Thus, our study suggested that the problem appeared to be temporary.

In the current study, we observed an average of seven flank lesions per pig, which is more than that reported by van Staaveren et al. (5) in finisher pigs namely <1 mild and 2 severe flank lesions per pig. In addition, van Staaveren et al. (7) observed that 0.4% of weaners at ages of 8–13 weeks had lesions. The difference might owe to the fact that the current study observed lesions from close distance instead of scanning the group of pigs outside of their pen.

A further explanation for the higher level of flank lesions in the present study as compared to that of van Staaveren et al. (5, 7) might be that the pigs in the current study were older at weaning (4 vs. 9 weeks). Weaning at 9 weeks is considerably older than in common commercial pig production. In the current study, flank biting behavior appeared to peak at 2–4 weeks after weaning, while lesions were more frequently observed in older pigs in the study of van Staaveren et al. (7). Thus, it is difficult to evaluate the effect of piglet age and that of weaning age in the light of the current scientific literature. A possible reason for this finding might be that even though weaning might cause a temporary increase in flank biting behavior, piglets are normally still very small at this age, and do not easily cause damage to the skin by manipulating each other. However, as the pigs in the current study were older, and thus probably stronger at the time of weaning, the increase in flank manipulation after weaning more easily resulted in skin damage, and thus the lesions.

While the etiology of flank lesions is not clear, our results suggest that biting, nosing, or snout manipulation could be a contributing factor. In the current study, the manipulative behavior appeared to increase at the same time when the flank lesion numbers were the highest, during weeks 2–4 after weaning. Actual biting events were only seldomly observed in our study (results not shown), while nosing was the predominant form of manipulative behavior. This supports the hypothesis proposed by Mirt (9) that flank lesions are due to a combination of a slight trauma caused by other pigs and a bacterial infection, instead of being due to mechanical damage from biting only.

Biting and other injurious social behaviors occur sporadically, creating difficulties in the observation of these situations. van Staaveren et al. (5) recorded 0.24 flank-directed damaging behaviors per finisher pig per hour. Diana et al. (12) observed 1–60 flank biting behaviors per hour per pen in weaner pigs. Due to the large number of animals in the pen, scan sampling was chosen as a feasible method. The current study included all nosing and biting events during scan sampling of a large pen and a group of pigs.

While very little is known about flank manipulation, more information is available on belly nosing, which appears to be somewhat similar behavior. Both behaviors are directed to the middle part of the trunk and involve nose contact. Belly nosing behavior is considered to be linked with general nosing activity (14, 15), peaking 2–3 weeks after weaning in piglets weaned at 12 or 21 days of age (16). Piglets weaned at the age of 4 weeks exhibited a peak in belly nosing frequency 2 weeks after weaning (17, 18). The increase in disturbing behaviors after weaning perhaps indicates negative effects on welfare caused by abrupt weaning. Belly nosing behavior increased in a stressful environment (with unfamiliar conspecifics, no straw, and less space allowance) after weaning (19). Belly nosing piglets tended to grow slower than others after weaning and are suggested to be affected by nutritional needs, which motivate massage-like behavior (15). On the other hand, belly nosing behavior intermingled in sequences with social interaction, suggesting that the motivational background is related to social needs (14). Some piglets perform more belly nosing and some less, but it is generally performed by the majority of piglets (14). However, it is unclear whether belly nosing and flank biting are different behaviors linked with each other or perhaps behaviors with the same motivational backgrounds.

The number of linear lesions increased during the first week after weaning, paralleling the increase in circular lesions. These linear lesions are assumed to be caused by fighting between pigs for resources such as feeder space after weaning from the sow. The number of linear lesions indeed indicated that better gaining weaners engaged in more fighting than pigs gaining weight more slowly, as fighting activity is correlated with more lesions (20).

Tail lesions were very rare and mild, and their occurrence displayed no marked temporal patterns, in contrast to the other lesion types, which suggests that the risk factors for tail lesions are not the same as for circular flank lesions. However, the housing system on the present farm includes several features that are known to reduce the risk of tail biting, including straw (21), wooden logs (22), and a low animal density (23).

In the current study, lesion occurrence was related to the whiter trunk coloration of the pigs. It is possible, that this was merely due to the white trunk coloration making lesions more visible, not to trunk coloration being an actual risk factor for lesions. However, color of the skin could potentially also be linked to personality traits (24). However, as trunk coloration has not been reported in earlier studies, the significance of it is difficult to assess. We thus recommend reporting skin color in connection with lesion scoring results.

## 5. Conclusion

Flank lesions increased after weaning at the age of 9 weeks and peaked during the 3rd week after weaning in this group. The emergence of flank lesions is possibly connected to weaning-related factors.

## Data availability statement

The raw data supporting the conclusions of this article will be made available by the authors, without undue reservation.

## Ethics statement

The animal study was reviewed and approved by National Animal Experiment Board, Complied European Directive 2010/63. Written informed consent was obtained from the owners for the participation of their animals in this study.

## Author contributions

MN, H-LK, and AV contributed to conception and design of the study. H-LK organized the database and wrote sections of the manuscript. MN performed the statistical analysis and wrote the first draft of the manuscript. All authors contributed to manuscript revision, read, and approved the submitted version.
